# Improved and Flexible HDR Editing by Targeting Introns in iPSCs

**DOI:** 10.1007/s12015-022-10331-1

**Published:** 2022-01-28

**Authors:** Juan Fu, Ya-Wen Fu, Juan-Juan Zhao, Zhi-Xue Yang, Si-Ang Li, Guo-Hua Li, Zi-Jun Quan, Feng Zhang, Jian-Ping Zhang, Xiao-Bing Zhang, Chang-Kai Sun

**Affiliations:** 1 Liaoning Provincial Key Laboratory of Cerebral Diseases, Institute for Brain Disorders, Dalian Medical University, Dalian, 116044 China; 2 Department of Obstetrics and Gynecology, The First Affiliated Hospital of Dalian Medical University, Dalian, 116044 China; 3 State Key Laboratory of Experimental Hematology, National Clinical Research Center for Blood Diseases, Haihe Laboratory of Cell Ecosystem, Institute of Hematology & Blood Diseases Hospital, Chinese Academy of Medical Sciences & Peking Union Medical College, Tianjin, 300020 China; 4 Research & Educational Center for the Control Engineering of Translational Precision Medicine (R-ECCE-TPM), School of Biomedical Engineering, Faculty of Electronic Information and Electrical Engineering, Dalian University of Technology, Dalian, 116024 China

**Keywords:** iPSCs, CRISPR, HDR, intron

## Abstract

Highly efficient gene knockout (KO) editing of CRISPR–Cas9 has been achieved in iPSCs, whereas homology-directed repair (HDR)-mediated precise gene knock-in (KI) and high-level expression are still bottlenecks for the clinical applications of iPSCs. Here, we developed a novel editing strategy that targets introns. By targeting the intron before the stop codon, this approach tolerates reading frameshift mutations caused by nonhomologous end-joining (NHEJ)-mediated indels, thereby maintaining gene integrity without damaging the non-HDR-edited allele. Furthermore, to increase the flexibility and screen for the best intron-targeting sgRNA, we designed an HDR donor with an artificial intron in place of the endogenous intron. The presence of artificial introns, particularly an intron that carries an enhancer element, significantly increased the reporter expression levels in iPSCs compared to the intron-deleted control. In addition, a combination of the small molecules M3814 and trichostatin A (TSA) significantly improves HDR efficiency by inhibiting NHEJ. These results should find applications in gene therapy and basic research, such as creating reporter cell lines.

**Supplementary Information:**

The online version contains supplementary material available at 10.1007/s12015-022-10331-1.

## Introduction

Induced pluripotent stem cells (iPSCs) provide an ideal source for cell replacement therapy and regenerative medicine due to their unlimited self-renewal and multidirectional differentiation ability [1]. However, the realization of the full therapeutic potential of human iPSCs requires further development of approaches to generate gene-modified or disease gene-corrected cells.

The clustered regularly interspaced short palindromic repeats (CRISPR)–Cas9 system has become a valuable tool for gene editing, from manipulating human cell genomes to creating gene-modified animal models. Its simplicity and robustness make it stand out from other genome editing technologies [2–4]. The CRISPR–Cas9 genome editing system, developed from the adaptive immune system of bacteria and archaea, consists of a Cas9 nuclease and a single-guide RNA (sgRNA) [5]. In this study, we used *Streptococcus pyogenes* (Sp) Cas9, the earliest and most commonly used Cas protein, for genome editing [3, 4, 6]. SpCas9 nuclease introduces double-stranded DNA breaks (DSBs) 3 bp upstream of the NGG protospacer adjacent motif (PAM) under the guidance of sgRNA. Since broken DNA is a dangerous signal for cells and causes severe cytotoxicity [7, 8], the DNA repair machinery is recruited and activated rapidly to promote DNA ligations through DNA repair pathways. Two of the main DSB repair pathways in mammalian cells are nonhomologous end-joining (NHEJ) and homologous recombination (HR), in which NHEJ generates a KO phenotype by introducing small insertions or deletions (indels) and is considered error-prone. In contrast, precise gene KI is a templated editing process guided by homology-directed repair (HDR) donors, which requires the presence of a recombination donor flanked with right and left homology arms (HAs) to generate precise editing outcomes.

Reporter cell lines are valuable tools for tracing cell lineages, visualizing corresponding gene expression levels, and investigating protein localization and function. CRISPR genome editing technology enables tagging a fluorescent gene to an endogenous gene to report the tagged gene. However, lower signal intensity is often a limiting factor, especially in genes with low expression. Developing brighter reporter cell lines, especially clinically relevant iPSCs and other cell lines, will provide an improved research tool.

Precise HDR gene editing has difficulty achieving acceptable efficiency for clinics even after CRISPR–Cas9 creates a DSB at the targeting site [9–11]. However, using a double cut donor plasmid design, we achieved a 5- to 10-fold increase in HDR KI efficiencies [12]. In addition, the transient overexpression of BCL-XL considerably increases genome editing efficiency in human iPSCs by enhancing cell survival after electroporation of editing plasmids [13]. Therefore, we used double-cut plasmid donor and BCL-XL plasmid electroporation for editing iPSCs in this study.

To establish a fluorescent reporter cell line, one straightforward strategy is to add a tag at the stop codon of a gene. However, the versatility of this method is limited because biallelic editing is much less efficient than monoallelic editing. Precise KI on one allele is often accompanied by indels on another allele, which may disrupt gene structure and cause gene deletion. Some groups have reported accurate gene integration by CRISPR–Cas9 intron targeting, which maintains the integrity of targeted endogenous genes and increases the rate of in-frame insertion compared to exon-based targeting [14–16]. In addition, multiple sgRNAs can often be identified to target an intron. In eukaryotic genomes, the precision and complexity of intron removal during pre-mRNA maturation requires the integrity of introns and other complex factors, and the disruption of standard splicing patterns can be a mechanism to downregulate the expression of a gene and cause diseases [17]. In addition, the Encyclopedia of DNA Elements (ENCODE) project has reported that introns often harbor enhancers for delicate regulation of gene expression [18]. Consequently, the realization of highly expressed fluorescent protein KI requires the integrity of genomic cis-elements, including in-frame fused exons and introns with complete structures.

In this study, we first conducted an intron-targeting KI strategy using an intron-deleted HDR donor. We achieved high efficiencies, but the fluorescent reporter expression intensity was lower than in cells edited with a mutated intron-containing HDR donor. Consequently, we developed an artificial intron KI strategy to generate edited cells efficiently without reducing the expression intensity of the fluorescent reporter genes. We also found that using an enhancer-containing intron may increase the reporter expression levels.

## Materials and Methods

### sgRNA Design

The CHOPCHOP website (http://chopchop.cbu.uib.no) [19, 20] was used to design appropriate sgRNAs targeting the last introns of human *OCT4*, *EEF1A1*, and *GAPDH*. Sequences of all the sgRNAs used in this study are listed in Supplementary Table 1.

### Plasmids Construction

All plasmids expressing Cas9, BCL-XL, sgRNAs, or mNeonGreen HDR donors were constructed with a NEBuilder HiFi DNA Assembly Kit (New England Biolabs) as described previously [12, 13]. In short, all the vector components were amplified from human gDNA or plasmids in our lab by PCR using KAPA HiFi polymerase (KAPA Biosystems) and purified using the GeneJET Gel Extraction Kit (Thermo Fisher Scientific). The PCR products were then assembled using the NEBuilder HiFi DNA Assembly kit following the manufacturer’s instructions. Multiple colonies were chosen for Sanger sequencing (MCLAB) to identify the correct clones. All the vectors were verified by Sanger sequencing. The mNeonGreen HDR donor (without intron) consisted of a mNeonGreen reporter protein flanked by ~600 bp homologous arms (HAs) and sgDocut (donor cut) recognition sequences. The introns will be deleted after HDR; thus, the HDR-edited targets will not be recognized by the sgRNAs.

### Constructions of Mutant or Artificial Intron-Containing HDR Donors

The double-cut donor plasmids used in this study were generated using the NEBuilder HiFi DNA Assembly kit (New England Biolabs), as detailed above. The self-cleaving E2A linker fragment is located between the modified intron-exon (the STOP codon on the last coding exon was deleted) and mNeonGreen or Crimson reporter, flanked by HAs and sgDocut recognition sequences [12]. For knockin with an endogenous intron-containing donor, a stretch of point mutation was introduced into the HDR donor’s intron. All the vectors were verified by Sanger sequencing. All the mutant wild-type introns or artificial introns are shown in Figs. 3a, b, and 4a. All the sequences of plasmid HDR donors used in this study are listed in Supplementary File 1.

### Human iPSC Culture

iPSC lines were generated from anonymous adult donors by peripheral blood (PB) reprogramming using episomal vectors expressing OCT4, SOX2, MYC, KLF4, and BCL-XL [21–23]. The iPSCs used in this study have been published previously [13, 24]. hiPSCs were grown under feeder-free conditions and maintained on tissue culture-treated 6-well plates (BD) coated with 1% Matrigel (Corning) in fresh mTeSR™E8 medium (StemCell Technologies). Cells at 60–70% confluency were passaged with 0.5 mM EDTA in PBS. Cells were cultured in a humidified atmosphere with 5% CO_2_ at 37 °C, and the medium was changed daily with fresh mTeSR™ E8 medium.

### Electroporation of iPSCs

For genome editing of iPSCs, cells were transfected by electroporation using the Amaxa Human Stem Cell Nucleofector® Kit 2 (Lonza) and the program B-016 on a Lonza nucleofector 2b. Briefly, 70 μl electroporation solution was prepared for each reaction, including 57.4 μl of the nucleofector solution, 12.6 μl of the supplement, and plasmids. Generally, 1 μg of Cas9 plasmids, 0.5 μg of sgRNA plasmids, 0.5 μg of sgDocut plasmids (for cutting pDonor in some experiments), and 1 μg of pDonor plasmids were used. In addition, we also used 0.5 μg of BCL-XL plasmid to improve iPSC survival [13]. iPSCs at 60–70% confluency were dissociated with the addition of 400 μl of Accutase, gently pipetted three times, and filtered with a 70 μm filter to obtain a single-cell suspension. Approximately 1–1.5 × 10^6^ cells were washed with DPBS (Gibco) and centrifuged at 400×g for 5 min, and the supernatant was carefully aspirated by vacuum. The cells were then resuspended in electroporation solution and carefully transferred into the cuvette. After electroporation, the cuvette was incubated at 37 °C for ∼5 min to improve cell survival [25]. The cells were then seeded onto Matrigel-coated plates in mTeSR™ E8 medium with 10 μM ROCK inhibitor Y-27632 (Millipore). Cells were gently handled during each step to reduce physical damage to the cells. One day later, cultures were fed fresh mTeSR™ E8 medium without any small molecules or drugs.

### Flow Cytometry

Flow cytometry was performed to determine reporter gene HDR efficiency, as described previously [13]. iPSCs were dissociated with Accutase and acquired on a BD FACS Canto II flow cytometer three days post-electroporation. For HDR-mediated KI of mNeonGreen or Crimson reporter into the target genes, the fluorescence-positive cell population was considered the HDR-edited cells. The FITC or APC channel was used to determine the proportion of mNeonGreen^+^ or Crimson^+^ cells. Electroporation without relevant sgRNA was carried out as a negative control, which showed few mNeonGreen^+^ or Crimson^+^ cells. The FACS data were analyzed using FlowJo™ 10.

### Illumina Deep Sequencing and Data Analysis

iPSCs edited without HDR donors were harvested three days after nucleofection for editing efficiency detection. Approximately 2 × 10^5^ cells were harvested for genomic DNA extraction using 10–20 μl digestion buffer, which consisted of 100 mM NaCl, 10 mM Tris pH 8, 5 mM EDTA, 0.5% Tween 20 (Sigma), and 1% proteinase K (ABM; 10 mg/ml). The mixtures were treated at 56 °C for 60 min, followed by 95 °C for 10 min. After a short spindown, one μl of the supernatant was used for PCR amplification. Genomic regions of interest were amplified by PCR using KAPA HiFi DNA polymerase. The thermal cycler program for primary PCR was as follows: 98 °C for 1 min, followed by 98 °C for 5 s, 64 °C for 5 s, 68 °C for 5 s, and 72 °C for 30 s for 30 cycles. PCR amplifications were verified by electrophoresis on 1% agarose gels. Then, 100 ng of barcoded PCR products from each sample were pooled for sequencing using Illumina’s NovaSeq6000 System (Novogene). Novogene constructed the library and acquired raw data. The 150-bp paired-end high-throughput sequencing reads were merged with FLASH [26], followed by demultiplexing using the Barcode Splitter Python script (https://pypi.org/project/barcode-splitter/). The indel efficiencies were analyzed with the docker version of CRISPResso2 [27], which returns many results, including the number of reads, details of editing alleles, and editing efficiencies.

### Nanopore Sequencing and Data Analysis

iPSCs edited with HDR donors were harvested three days after nucleofection. Genomic DNA was extracted using the Gentra Puregene Blood Kit (Qiagen). Genomic regions of interest were amplified by KAPA HiFi DNA polymerase. The PCR cycling condition was 98 °C for 1 min, followed by 98 °C for 10 s, 64 °C for 15 s, and 72 °C for 90 s for 30 cycles. PCR amplifications were verified by electrophoresis on 1% agarose gels. An equal amount of barcoded PCR products of different targets were pooled for nanopore sequencing using PromethION (ONT, UK) at Novogene. Albacore (version 2.3.1, Oxford Nanopore Technologies) transformed raw fast5 data into bases and quality scores. Then, we processed the data as described previously [28, 29]. In brief, sequencing adapters were removed by Porechop [30] (version 0.2.4) and then processed with Seqkit to grep for individual reads of PCR products. Next, we used Minimap2 [31] (version 2.14) to align the fastq sequences to the reference fasta files (HDR sequences). The aligned bam files were visualized using IGV [32] (version 2.10.3).

### Small Molecules

Commercially available small molecules used in this study were trichostatin A (TSA) (Cayman; 89,730) and M3814 (MedKoo; 206,478). iPSCs were split into two culture wells after electroporation with editing plasmids to assess the effects of small molecules. Stock solutions of the HDAC inhibitor TSA and NHEJ inhibitor M3814 were prepared in dimethylsulfoxide (DMSO) (Sigma) and diluted to working concentrations before use. The parallel well with only DMSO (0.1%) served as a control. The medium was changed 24 h after the addition of small molecules.

### Statistics and Reproducibility

The *P* values for different groups were calculated and analyzed by paired Student’s *t* test. In all significance tests performed in the study, the data satisfied the normality criteria for *t* tests. *, *P* < 0.05; **, *P* < 0.01; ***, *P* < 0.001; ****, *P* < 0.0001; ns., not significant. Bar graphs in figures were plotted, and s.d. error bars were calculated using GraphPad Prism 8. Scatterplots of correlation and linear regression analysis were calculated using Pearson’s correlation coefficient.

## Results

### Generation of iPSC Reporter Cell Lines by CRISPR–Cas9 Intron Targeting

**Fig. 1:**
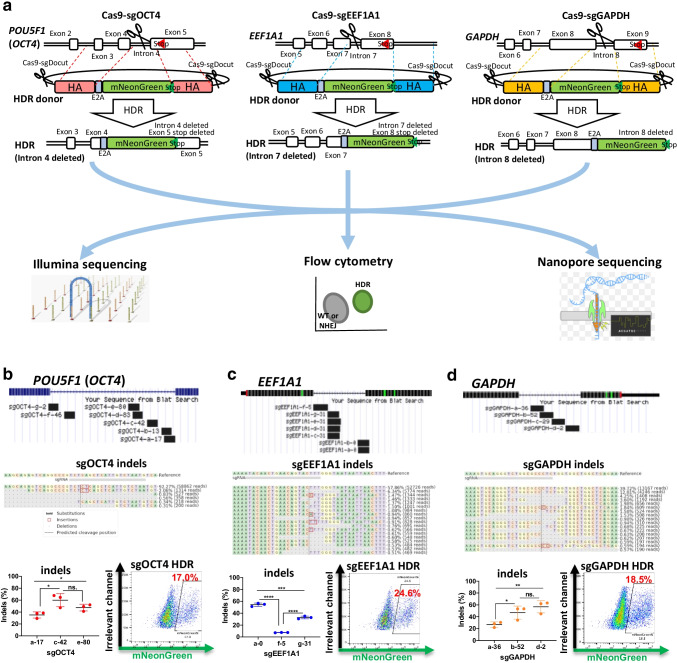
CRISPR–Cas9 intron editing in iPSCs. **a** Schematics of genome editing with Cas9-sgRNA plasmids and an HDR donor template. **b-d** Editing efficiencies and mNeonGreen HDR KI at *OCT4* (**b**), *EEF1A1* (**c**), and *GAPDH* (**d**) in iPSCs. Top, Schematics of targeting the intron with multiple sgRNAs. The number in the sgRNA ID indicates the distance between the cut site and the proximate intron-exon junction. Middle, Representative editing outcomes analyzed by CRISPResso2. Bottom, editing efficiencies of representative sgRNAs (left) and mNeonGreen HDR KI (right). HDR efficiencies were determined by FACS analysis three days after electroporation. Data are shown as the mean ± s.d. Unpaired two-sided Student’s *t* tests were conducted. ****P < 0.0001; ***P < 0.001; **P < 0.01; *P < 0.05; n.s., *P* ≥ 0.05

This study designed 18 sgRNAs that target the last introns of three commonly studied human genes (seven for *OCT4*, seven for *EEF1A1*, and four for *GAPDH*) (Supplementary Table 1). To tag these genes with a fluorescent protein, we conducted electroporation of iPSCs with Cas9-sgRNA and HDR double-cut donor plasmids. The HDR donor vectors contained E2A-mNeonGreen flanked by HAs omitting introns (Fig. 1a, top). We assessed the indel frequencies three days after electroporation by amplifying target sequences and Illumina sequencing. HDR efficiencies were determined by FACS analysis of mNeonGreen-positive cells. No mNeonGreen-positive cells were detectable in the negative controls, suggesting that mNeonGreen-positive cells were HDR edited (Supplementary Fig. S1). To consolidate this conclusion, we performed nanopore sequencing of representative KI samples and observed precise insertion of the reporter gene (Fig. 1a).

We designed sgRNAs to target the intron before the stop codon. To identify the optimal sgRNAs, we first compared the KO editing (indel) efficiencies of different sgRNAs (Fig. 1b-d, top). Editing efficiencies and patterns were assessed with CRISPResso2 [27] (Fig. 1b-d, middle). We observed variable indel and HDR efficiency for each sgRNA.

### Relative HDR Efficiencies Negatively Correlate with the Distances from the Cut Site to the Intron-Exon Junction

**Fig. 2:**
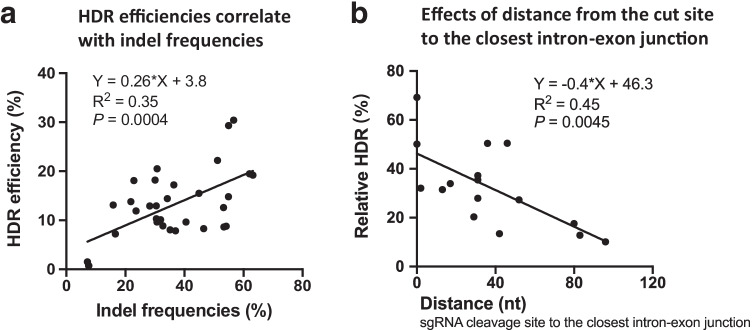
HDR efficiencies negatively correlate with the distance from the cut site to the intron-exon junction. **a** HDR efficiencies correlate with indel frequencies. n = 32. **b** Relative HDR efficiencies negatively correlate with the distance from the cut site to the closest intron-exon junction. The relative HDR efficiencies are defined as absolute HDR efficiencies divided by indel frequencies. n = 16

We next analyzed the relationship between KO and KI efficiencies. As expected, Pearson linear regression analysis showed that HDR editings were proportional to indel efficiencies (*R*^*2*^ = 0.35, *P* = 0.0004) (Fig. 2a), suggesting that the sgRNA targeting ability largely dictates HDR efficiency.

In our design, cleavage at the intron leaves stretches of sequences that mismatch homologous sequences on the HAs. We speculate that the length of mismatch sequences might negatively affect HDR editing. Therefore, we conducted a correlation analysis between the relative HDR and the distance from the sgRNA cleavage site to the closest intron-exon junction. As shown in Fig. 2b, linear regression analysis using aggregated data showed that the relative HDR efficiencies negatively correlated with the distance from the intron-exon boundary (*R*^*2*^ = 0.45, *P* = 0.0045) (Fig. 2b). These results suggest that targeting sequences in the proximity of intron-exon junctions will lead to high-level relative HDR editing in intron-targeting applications.

### A Reporter Gene Knock-in without Deleting the Targeted Intron

**Fig. 3:**
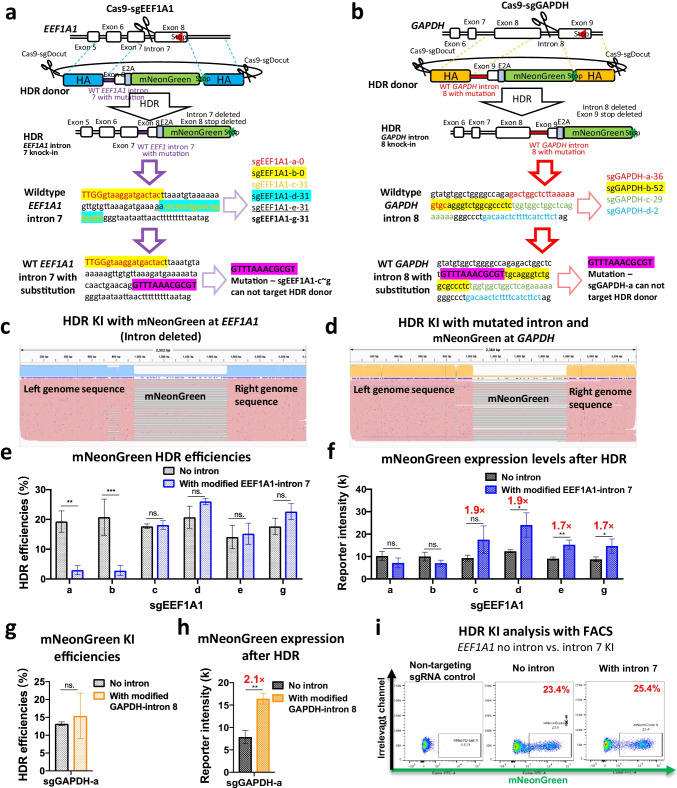
A reporter gene knock-in without deleting the target intron. **a, b** Schematics of mNeonGreen HDR knockin at *EEF1A1* intron 7 (**a**) and *GAPDH* intron 8 (**b**) in iPSCs. The target sequences of used sgRNAs are highlighted. HDR donors with *EEF1A1* or *GAPDH* introns were mutated to avoid cleavage by certain sgRNAs. The purple highlight shows the substitution. **c, d** IGV visualization of mNeonGreen HDR KI at *EEF1A1* (**c**) and *GAPDH* (**d**). **c** The use of no-intron donor leads to the deletion of intron and insertion of mNeonGreen after HDR editing. **e, f** HDR KI efficiencies (**e**) and mNeonGreen intensity (**f**) after HDR editing with or without the *EEF1A1* intron. The a-g in the X-axis represent a series of sgRNAs that target different locations at *EEF1A1* in (**a**). Note: cleavage of the HDR donor with sgEEF1A1-a and b leads to low KI levels (**e**). n = 3 independent biological repeats. **g, h** mNeonGreen intensities (expression levels) after editing with donors with or without introns. n = 3 independent biological repeats. (**i**) Representative FACS panels after HDR editing using donors with or without introns 7 3 days after electroporation. Data are shown as the mean ± s.d. Unpaired two-sided Student’s *t* tests were conducted. **P < 0.01; *P < 0.05; n.s., *P* ≥ 0.05

In the above studies, the mNeonGreen reporter was fused with the open reading frame by self-cleaving peptide E2A, and the intron was deleted after HDR-mediated KI (Fig. 1a). However, intron deletion may lead to decreased gene expression. As such, we modified the exon-E2A-mNeonGreen HDR donor by including the previously deleted intron and substituting the sgRNA targeting sequences with a 12-bp mismatch fragment to prevent cleavage of the HDR donor by the sgRNA (Fig. 3a-b). However, this strategy is only applicable to a limited number of sgRNAs. For instance, the substitutive exogenous fragments did not prevent cleavage by two sgEEF1A1s and three sgGAPDHs (Fig. 3a-b).

To validate the genetic changes after CRISPR-mediated modification of intron-exon-mNeonGreen KI, we PCR-amplified an ~1.6 kb region flanking the Cas9-sgRNA target sites at *EEF1A1* and *GAPDH*. The PCR products were sequenced and visualized by IGV [32], showing indels and insertions of ~700 bp fragments at *EEF1A1* and *GAPDH* (Fig. 3c-d) (including the modified endogenous intron and mNeonGreen, the HDR reference sequence is ~2.3 kb in length). We also determined HDR efficiencies and mNeonGreen expression levels by flow cytometry. sgEEF1A1-f was excluded from further analysis because of its low targeting efficiency (Fig. 1c, bottom left). Compared with the intron-deleted donor template, the *EEF1A1*-intron 7-containing donor showed similar HDR efficiencies (Fig. 3e, i) but significantly increased the mNeonGreen expression levels by ~2-fold (Fig. 3f, sgEEF1A1-c to -g). The HDR efficiencies of sgEEF1A1-a and -b showed striking decreases due to sgRNA-mediated cleavage of template plasmids and HDR alleles (Fig. 3a). Similarly, the intron-containing donor did not increase the HDR efficiency at *GAPDH*, whereas it showed a 2-fold increase in reporter gene expression (Fig. 3g-h). Careful examination of the genome sequence revealed that these introns contain an enhancer-like element (Supplementary Fig. S2). These data demonstrate that intron deletion after editing may decrease gene expression levels, whereas adding back a mutated intron will increase the reporter gene expression levels.

### HDR Editing with an Artificial Intron-Containing Donor Template

The previous design must construct multiple mutated intron-containing HDR donor plasmids to screen the best sgRNA. To simplify the workflow, we decided to replace the endogenous intron with an artificial intron from another gene. We constructed the *OCT4*-Crimson HDR donor that contains *EEF1A1*-intron 7. We chose sgOCT4-a in the following studies, considering the targeting efficiency and relative HDR ratio (Figs. 1b and 2b).

**Fig. 4:**
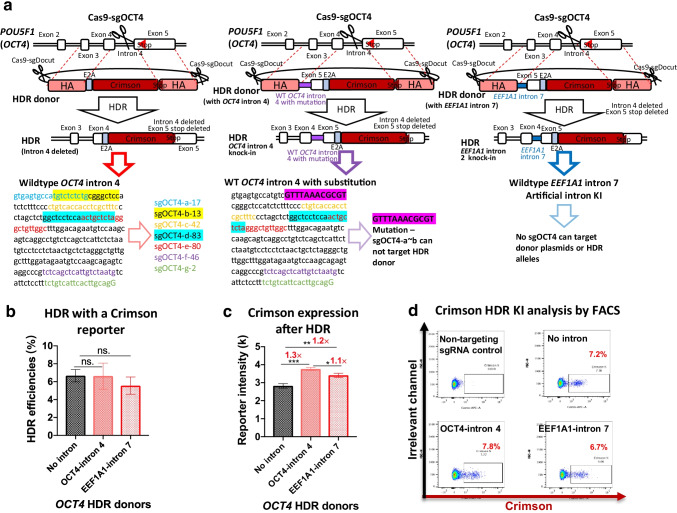
HDR editing with an artificial intron-carrying donor increases reporter expression levels. **a** Schematics of the *OCT4-*Crimson HDR donor plasmid with no intron as a control (left) or with *OCT4* intron 4 (middle) or *EEF1A1* intron 7 (right) in iPSCs. The target sequences of the used sgRNAs highlight that *OCT4* intron 4 in the donor template is mutated to avoid cleavage by certain sgRNAs. The purple highlight shows the substitution (middle). **b, c** KI efficiencies (**b**) and reporter intensity (**c**) after editing with HDR donors with no intron or with *OCT4* intron 4 (self-intron) or natural *EEF1A1* intron 7 (artificial intron) in iPSCs. n = 3. **d** Representative FACS panels after HDR editing using donors with or without introns. HDR efficiencies were determined by FACS 5 days after electroporation. Data are shown as the mean ± s.d. Unpaired two-sided Student’s *t* tests were conducted. **P < 0.01; *P < 0.05; n.s., *P* ≥ 0.05

Next, we examined each donor’s HDR efficiencies and Crimson expression levels. Few Crimson-positive cells were detected in the negative controls (Fig. 4d). Consistent with the previous conclusions, the *OCT4*-intron 4-containing donor significantly increased the expression intensity by 1.3-fold (Fig. 4b-c). These data consolidate the conclusion that reconstruction of gene integrity leads to improved gene expression. The artificial-intron-containing HDR template showed a slightly higher expression level (1.2-fold) (Fig. 4b-c) than the intron-deleted donor. In the three HDR KI strategies, the donor template that can best restore the natural structure of the target gene locus showed the best reporter gene HDR KI (Fig. 4b-d). Taken together, this versatile and straightforward artificial intron-containing HDR donor is inferior to the endogenous-intron-containing template but performs better than the intron-deleting strategy.

### The Combination of M3814 and Trichostatin A Increases Intron-Targeting HDR Editing Efficiencies

**Fig. 5:**
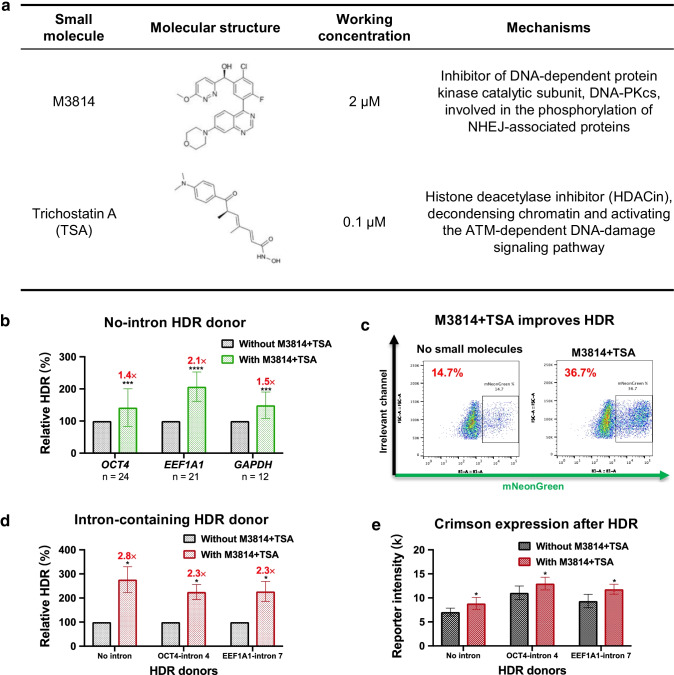
M3814 and trichostatin A increase intron-targeting HDR editing efficiencies in iPSCs. **a** Molecular structures, working concentrations, and mechanisms of action of M3814 and trichostatin A (TSA). **b** M3814 and TSA enhance HDR efficiencies at *OCT4*, *EEF1A1*, and *GAPDH* in iPSCs with mNeonGreen HDR donors. Paired two-sided Student’s *t* tests were conducted. ****P < 0.0001; ***P < 0.001. **c** Representative FACS panels after editing without or with M3814 and TSA. **d, e** HDR efficiencies (**d**) and Crimson intensities (reporter gene expression levels) (**e**) after editing with HDR donors containing no intron, *OCT4* intron 4, and natural *EEF1A1* intron 7 in the absence or presence of small molecules. HDR efficiencies were determined by FACS 5 days after electroporation for the APC signal. n = 3. Data are shown as the mean ± s.d. Unpaired two-sided Student’s *t* tests were conducted. **P < 0.01; *P < 0.05; n.s., *P* ≥ 0.05

NHEJ is the predominant and fast-acting pathway to repair CRISPR-mediated DSBs and outcompetes other editings [24, 33]. Therefore, NHEJ inhibitors improve HDR efficiency [34–36]. One of the most effective NHEJ inhibitors is M3814, which strikingly improves HDR by blocking NHEJ [24, 37]. Additionally, we have demonstrated that HDAC inhibitors promote gene editing efficiencies at closed and open chromatin loci [38]. Thus, we speculated that the robust small-molecule combination M3814 and trichostatin A (TSA) should enhance HDR in our intron editing system (Fig. 5a).

We first investigated the effects of the M3814 + TSA combination on HDR efficiencies using no-intron HDR donors (Fig. 1a). Next, we collected mNeonGreen HDR editing data from 57 edited samples to assess statistical significance at *OCT4*, *EEF1A1*, and *GAPDH*. The relative HDR efficiency was computed by comparing the percentages of mNeonGreen-positive cells in the presence or absence of the two agents (Fig. 5b). The combination M3814 + TSA performed well at all tested loci, increasing the HDR efficiencies by up to ~2.5-fold (Fig. 5c).

We further investigated the small molecule mix using intron-containing HDR donors. As expected, M3814 and TSA increased the HDR efficiencies in all types of HDR donors and showed a significant improvement (up to 2.8-fold) (Fig. 5d). Additionally, the combination slightly increased the average Crimson reporter expression by ~1.2-fold, which might result from a greater proportion of cells being edited biallelically. (Fig. 5e). These data further consolidate the conclusion that intron deletion after HDR may decrease gene expression, and adding back a mutated or artificial intron will increase the reporter gene expression levels.

Expression of the reporter gene after editing was also visualized using fluorescent microscopy (Supplementary Fig. S3a). We also found that unedited and edited iPSCs were morphologically similar one week after transfection of editing plasmids (Supplementary Fig. S3b). To investigate whether editing affects the cellular phenotypes of the iPSCs, we conducted staining of TRA-1-60, a stemness surface marker, in untreated and edited iPSCs and observed no difference in expression levels (Supplementary Fig. S3c). These data suggest that editing at these loci does not negatively affect iPSCs.

## Discussion

Herein, we describe a novel intron-targeting HDR knock-in approach by using an artificial intron-containing donor template. Small indels mediated by NHEJ introduced into the intron are less likely to affect target gene function or induce gene deletion. In addition, intron targeting also theoretically increases the rate of in-frame insertion compared to exon-based targeting [16]. Furthermore, an intron retention strategy avoids the disruption of cis-regulatory elements such as proximal or distal enhancers at the intron regions, thus leading to high-level expression of reporter genes. We showed an up to 2-fold increase in reporter gene expression (Fig. 3f, h). Therefore, these intron knock-in strategies have two advantages: (1) inserted exogenous genes can faithfully recapitulate the expression pattern of targeted endogenous genes; (2) one can achieve higher expression levels of the fluorescent reporter gene.

After CRISPR-mediated dsDNA cleavage, sgRNAs and contextual sequences primarily determine the indel frequencies, editing patterns, and HDR efficiencies (Figs. 1b-d and 2a). However, we also observed an interesting phenomenon. The relative HDR efficiency was related to the target site on the intron, where the sgRNAs targeting sequences close to the adjacent exon showed better relative HDR (Fig. 2b). Editing with different intron-targeting sgRNAs with the same intronless HDR donor leads to the generation of two mismatch sequences with different lengths. After DSBs, the surrounding sequences survey for homology during HDR. A significant stretch of nucleotides that mismatches the donor template will negatively affect the homology search. Thus, the best DSB-mediated DNA gaps are in principle flanked by the exact homologous sequences for HDR. Our data suggest that it is favorable for HDR to use sgRNAs that generate HA-friendly DSBs or target the proximity of the intron-exon junction. At least one arm has the shortest mismatched sequence in such a case. Another laboratory reported that the nonhomologous sequence of the donor reduced the efficiency of transgene integration [39], lending support to our conclusion.

A primary feature of most eukaryotic genes is that they are interrupted by introns, which are removed during transcription to create mRNAs with intact open reading frames. For example, the average human gene contains eight exons and seven introns, producing an average of three or more alternatively spliced mRNA isoforms [40]. Introns can influence and enhance initial transcription of the gene, editing and polyadenylation of the pre-mRNA, nuclear export, RNA stability and translation, and mRNA decay [41, 42]. In addition, multiple intron regions show enhancer-like features. Thus, it is not surprising that disruption or loss of introns can affect gene regulation and expression levels.

Based on the essential role of introns in the human genome, we modified our HDR strategy, that is, knocking in reporter genes without disrupting the intron, thus maintaining the integrity of the natural genes. As a result, the intron-containing donor showed HDR efficiencies similar to those of the no-intron donors (Figs. 3e, g and 4b). This result consolidates the conclusion that the editing efficiencies and HDR mainly depend on the sgRNA context and intron-exon junctions (Fig. 2). However, the intron-containing HDR donors significantly increased the fluorescent reporter expression levels (Figs. 3f, h, and 4c). We checked the targeted intron regions of these three genes on UCSC Genome Browser ENCODE Candidate Cis-Regulatory Elements (cCREs) and observed that the targeted intron regions have proximal or distal enhancer-like signatures (Supplementary Fig. S2). These associations can explain the improved fluorescent reporter expression levels after intron-containing HDR editing. To the best of our knowledge, we are the first to report a flexible artificial intron KI strategy that allows the screening of multiple sgRNAs. Although it does not show a significant improvement over the endogenous-intron-containing donor, it is still a handy choice for HDR editing to quickly establish a report line with high efficiency and expression levels.

M3814 and TSA increase HDR efficiency in many editing systems [24, 37, 38]. M3814 promotes HDR editing by strongly inhibiting NHEJ-mediated indels. As an effective HDAC inhibitor, TSA can decondensate chromatin, which increases the local concentration of sgRNAs, Cas9, and HDR donors. Thus, combined M3814 and TSA treatment strongly inhibits NHEJ and increases chromatin accessibility simultaneously. The addition of M3814 and TSA increased the apparent HDR efficiency by 2.8-fold in iPSCs (Fig. 5d).

In summary, we present a flexible and improved method using an artificial intron-containing HDR donor template to create iPSC reporter cell lines efficiently. Furthermore, targeting the intron decreases the possibility of disrupting the coding sequence. In addition, M3814, together with TSA, considerably increases HDR editing efficiencies.

## Supplementary Information

ESM 1 (PPTX 7134 kb)

ESM 2 (DOCX 27 kb)

ESM 3 (XLSX 10.9 kb)

## Data Availability

The authors declare that all data supporting the conclusions in this study are available within the article and its extended information files on reasonable requests. All Illumina generation and Nanopore sequencing data gzip files have been deposited in the GEO database under the accession code GSE155891.
