# Supplementary material for: Improved and Flexible HDR Editing by Targeting Introns in iPSCs
Source: Stem Cell Rev Rep. 2022 Jan 28;18(5):1822–33. doi: 10.1007/s12015-022-10331-1 (PMC9209395; doi:10.1007/s12015-022-10331-1)
Supplement: Supplementary file 1 — (PPTX 7134 kb) [file 12015_2022_10331_MOESM1_ESM.pptx]

## Slide 1
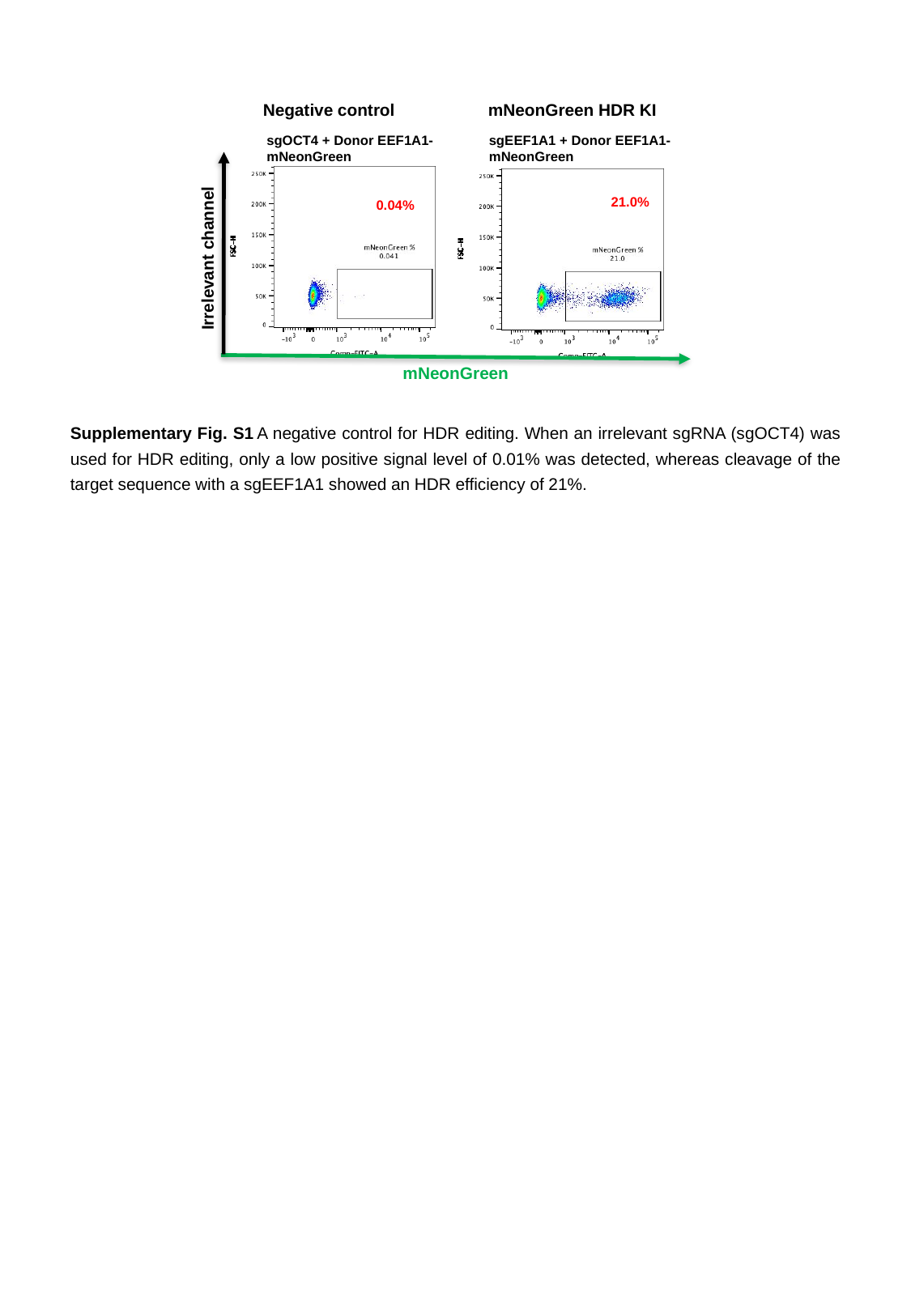

Negative control
mNeonGreen HDR KI
sgOCT4 + Donor EEF1A1-mNeonGreen
sgEEF1A1 + Donor EEF1A1-mNeonGreen
Irrelevant channel
mNeonGreen
21.0%
0.04%
Supplementary Fig. S1 A negative control for HDR editing. When an irrelevant sgRNA (sgOCT4) was used for HDR editing, only a low positive signal level of 0.01% was detected, whereas cleavage of the target sequence with a sgEEF1A1 showed an HDR efficiency of 21%.

## Slide 2
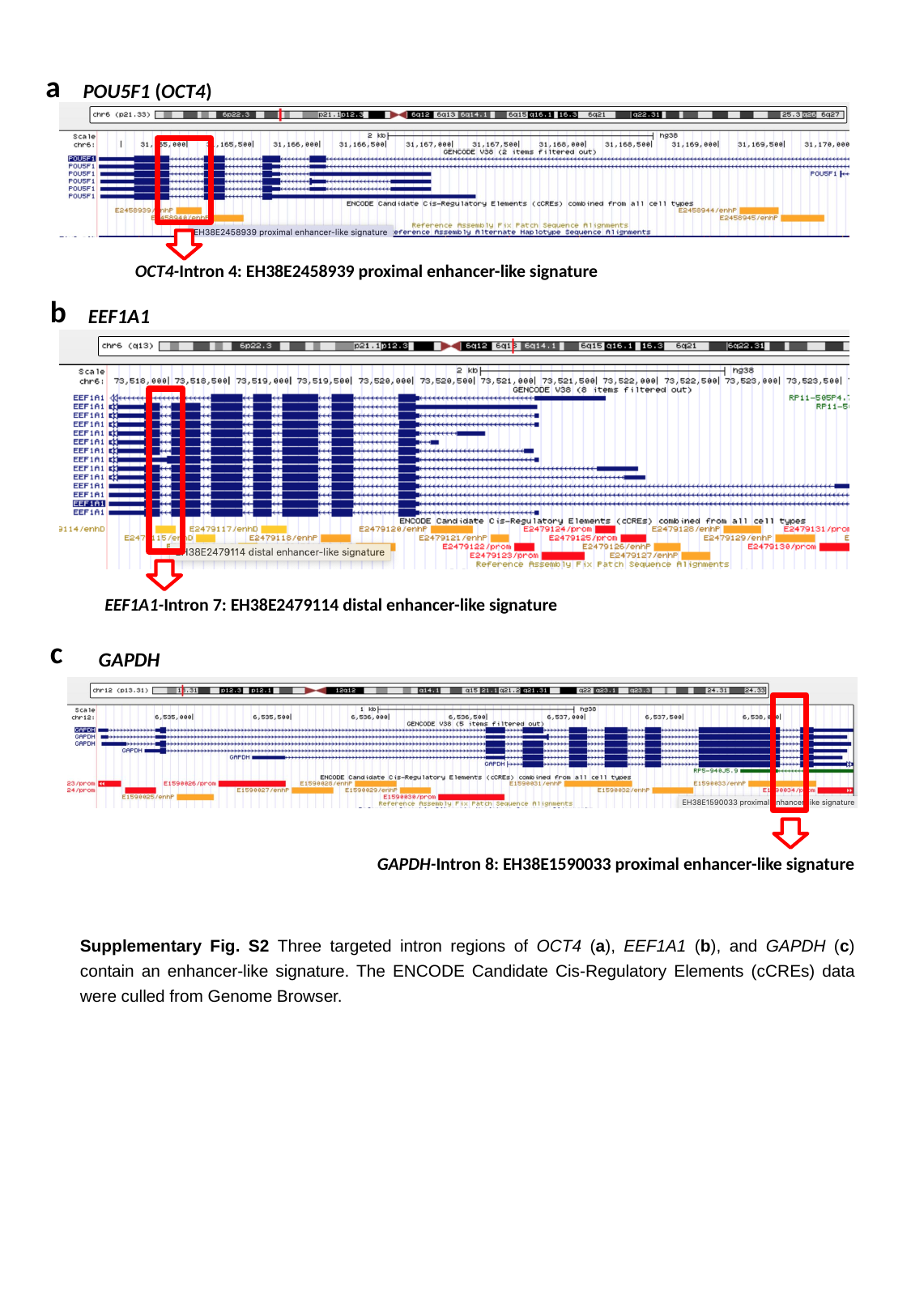

a
POU5F1 (OCT4)
OCT4-Intron 4: EH38E2458939 proximal enhancer-like signature
b
EEF1A1
EEF1A1-Intron 7: EH38E2479114 distal enhancer-like signature
c
GAPDH
GAPDH-Intron 8: EH38E1590033 proximal enhancer-like signature
Supplementary Fig. S2 Three targeted intron regions of OCT4 (a), EEF1A1 (b), and GAPDH (c) contain an enhancer-like signature. The ENCODE Candidate Cis-Regulatory Elements (cCREs) data were culled from Genome Browser.

## Slide 3
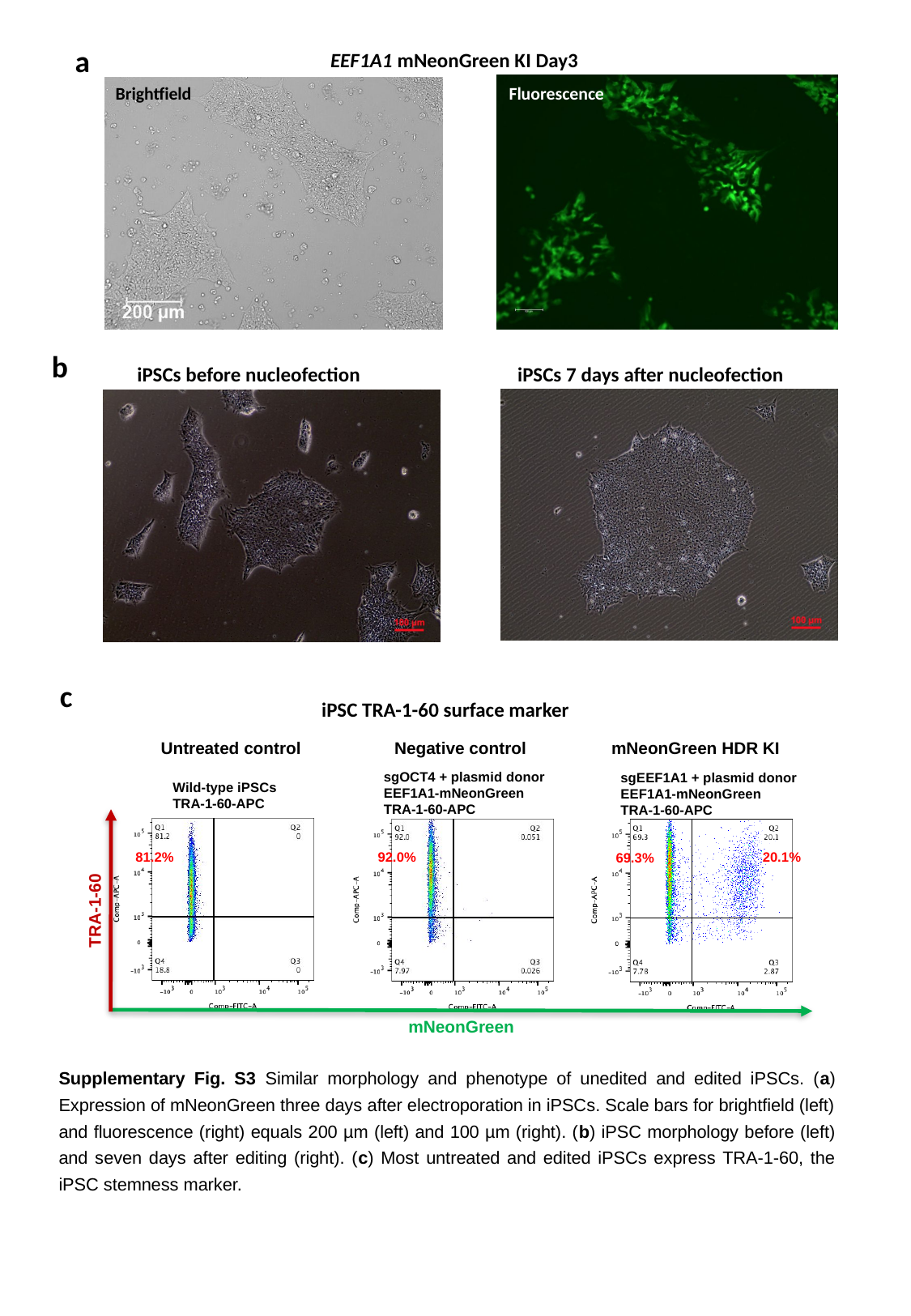

a
EEF1A1 mNeonGreen KI Day3
Brightfield
Fluorescence
b
iPSCs before nucleofection
iPSCs 7 days after nucleofection
c
iPSC TRA-1-60 surface marker
Untreated control
Negative control
mNeonGreen HDR KI
sgOCT4 + plasmid donor EEF1A1-mNeonGreen
TRA-1-60-APC
sgEEF1A1 + plasmid donor EEF1A1-mNeonGreen
TRA-1-60-APC
Wild-type iPSCs
TRA-1-60-APC
TRA-1-60
mNeonGreen
81.2%
92.0%
20.1%
69.3%
Supplementary Fig. S3 Similar morphology and phenotype of unedited and edited iPSCs. (a) Expression of mNeonGreen three days after electroporation in iPSCs. Scale bars for brightfield (left) and fluorescence (right) equals 200 µm (left) and 100 µm (right). (b) iPSC morphology before (left) and seven days after editing (right). (c) Most untreated and edited iPSCs express TRA-1-60, the iPSC stemness marker.
