# Supplementary material for: Improved and Flexible HDR Editing by Targeting Introns in iPSCs
Source: Stem Cell Rev Rep. 2022 Jan 28;18(5):1822–33. doi: 10.1007/s12015-022-10331-1 (PMC9209395; doi:10.1007/s12015-022-10331-1)
Supplement: Supplementary file 2 — (DOCX 27 kb) [file 12015_2022_10331_MOESM2_ESM.docx]

**HDR Donor Sequences**

**OCT4-E2A-mNeonGreen (Left HA-E2A-mNeonGreen-Right HA)**

tcagggtcacaagggccctgcgtgctccctcactttgcttctcttttgactggcctcccccagGGAAGGTATTCAGCCAAACGACCATCTGCCGCTTTGAGGCTCTGCAGCTTAGCTTCAAGAACATGTGTAAGCTGCGGCCCTTGCTGCAGAAGTGGGTGGAGGAAGCTGACAACAATGAAAATCTTCAGGAGgtaagggtgggagggggatacccggggaccttccctttcttggcctaatttccattgcttccatcactggctcgtagctctccgtctttggtgcagtggttctcagtgggatggagtgaaattcctcagttctgctgggataaggtccagagccaacccttccaggatcctgccttttcacaccaccacctggctctgctgacacatctagtcacagacccctgtgatgctgttactcagcaagtccaaagcttgcccttgtcacccccttcccacctgcacagATATGCAAAGCAGAAACCCTCGTGCAGGCCCGAAAGAGAAAGCGAACCAGTATCGAGAACCGAGTGAGAGGCAACCTGGAGAATTTGTTCCTGCAGTGCCCGAAACCCACACTGCAGCAGATCAGCCACATCGCCCAGCAGCTTGGGCTCGAGAAGGATGTGGTCCGAGTGTGGTTCTGTAACCGGCGCCAGAAGGGCAAGCGATCAAGCAGCGACTATGCACAACGAGAGGATTTTGAGGCTGCTGGGTCTCCTTTCTCAGGGGGACCAGTGTCCTTTCCTCTGGCCCCAGGGCCCCATTTTGGTACCCCAGGCTATGGGAGCCCTCACTTCACTGCACTGTACTCCTCGGTCCCTTTCCCTGAGGGGGAAGCCTTTCCCCCTGTCTCCGTCACCACTCTGGGCTCTCCCATGCATTCAAACCAGTGTACTAATTATGCTCTCTTGAAATTGGCTGGAGATGTTGAGAGCAACCCAGGTCCCATGGTGAGCAAGGGCGAGGAGGATAACATGGCCTCTCTCCCAGCGACACATGAGTTACACATCTTTGGCTCCATCAACGGTGTGGACTTTGACATGGTGGGTCAGGGCACCGGCAATCCAAATGATGGTTATGAGGAGTTAAACCTGAAGTCCACCAAGGGTGACCTCCAGTTCTCCCCCTGGATTCTGGTCCCTCATATCGGGTATGGCTTCCATCAGTACCTGCCCTACCCTGACGGGATGTCGCCTTTCCAGGCCGCCATGGTAGATGGCTCCGGATACCAAGTCCATCGCACAATGCAGTTTGAAGATGGTGCCTCCCTTACTGTTAACTACCGCTACACCTACGAGGGAAGCCACATCAAAGGAGAGGCCCAGGTGAAGGGGACTGGTTTCCCTGCTGACGGTCCTGTGATGACCAACTCGCTGACCGCTGCGGACTGGTGCAGGTCGAAGAAGACTTACCCCAACGACAAAACCATCATCAGTACCTTTAAGTGGAGTTACACCACTGGAAATGGCAAGCGCTACCGGAGCACTGCGCGGACCACCTACACCTTTGCCAAGCCAATGGCGGCTAACTATCTGAAGAACCAGCCGATGTACGTGTTCCGTAAGACGGAGCTCAAGCACTCCAAGACCGAGCTCAACTTCAAGGAGTGGCAAAAGGCCTTTACCGATGTGATGGGCATGGACGAGCTGTACAAGTAAGGTGCCTGCCCTTCTAGGAATGGGGGACAGGGGGAGGGGAGGAGCTAGGGAAAGAAAACCTGGAGTTTGTGCCAGGGTTTTTGGGATTAAGTTCTTCATTCACTAAGGAAGGAATTGGGAACACAAAGGGTGGGGGCAGGGGAGTTTGGGGCAACTGGTTGGAGGGAAGGTGAAGTTCAATGATGCTCTTGATTTTAATCCCACATCATGTATCACTTTTTTCTTAAATAAAGAAGCCTGGGACACAGTAGATAGACACACTTATCTTGGTTTGTCCTTCAGTTACTGAGGTAGGGATGGGAATATCCAATGCTCATACCCAAGTGACCCTGAAACTAAGGTGCCATTTACACTCCTTAAGGTCACACAACATCAGAGGGAGAGCTGGGATTGCAGCCAAGTTTATTTGTACAGGGCCCTGTGATAGGCTAGTTCCCAAAAGCCTGTGATGCAAGAACTTTTGCCCATAGACTCAGTCACCATGTAGCTGTTACCTGTTCAGAGCTGGCTTTTTGCTTTCCCACCCTACTCTGGAATTCTTAAATGGCTTTATACTTAGAAATCATCTTATTTCTGTTGAACCTAGATCACCCCAACCAGAAACTTCTATTAATACTTTGTGCTTTCTTGATACCAGGGTCTATTTGGTTTCCACTTAAGGTTTTTGCATACTCTGCCCATAAGTGACTCATTAGTTACTCAAGTTTTATTCCTGGCTCTGCCACTAGTTCATTAGGGGTCTTTGCCCCAGAGTCATTTCTTCCATTTTAAAAACTTGGGCTCATTAAATCTAGGTAGGAAAGGGCGGGTGTGGCAGGTTTTAATAGAACAGGTCAAGATAAGGCTTTATTTCTATAGAAATGATGCTTTGACAATAGTTTGGCTTGGTGTAAGGCTCACAAAAGAAAATCACATGTACCATGTGTGGGTTAAGCGGTTTGATTCACACTGAACCAGGCCAGCCCAGTTGCCCTCTGCTGTGTCCACCCGTGGAGTGGAGCTGTGTCACAGCCATCACACTGGTAAACTGCTGTAGCTGGTTTACCAGGCTTTCTCTTGCCCTGA

**EEF1A1-E2A-mNeonGreen (Left HA-E2A-mNeonGreen-Right HA)**

ggctgtaaacaaagttgaatttgagttgatagagtactgtctgccttcataggtatttagtatgctgtaaatatttttagGTATTGGTACTGTTCCTGTTGGCCGAGTGGAGACTGGTGTTCTCAAACCCGGTATGGTGGTCACCTTTGCTCCAGTCAACGTTACAACGGAAGTAAAATCTGTCGAAATGCACCATGAAGCTTTGAGTGAAGCTCTTCCTGGGGACAATGTGGGCTTCAATGTCAAGAATGTGTCTGTCAAGGATGTTCGTCGTGGCAACGTTGCTGGTGACAGCAAAAATGACCCACCAATGGAAGCAGCTGGCTTCACTGCTCAGgtaacaatttaaagtaacattaacttattgcagaggctaaagtcatttgagactttggatttgcactgaatgcaaatcttttttccaagGTGATTATCCTGAACCATCCAGGCCAAATAAGCGCCGGCTATGCCCCTGTATTGGATTGCCACACGGCTCACATTGCATGCAAGTTTGCTGAGCTGAAGGAAAAGATTGATCGCCGTTCTGGTAAAAAGCTGGAAGATGGCCCTAAATTCTTGAAGTCTGGTGATGCTGCCATTGTTGATATGGTTCCTGGCAAGCCCATGTGTGTTGAGAGCTTCTCAGACTATCCACCTTTGGGTCGCTTTGCTGTTCGTGATATGAGACAGACAGTTGCGGTGGGTGTCATCAAAGCAGTGGACAAGAAGGCTGCTGGAGCTGGCAAGGTCACCAAGTCTGCCCAGAAAGCTCAGAAGGCTAAACAGTGTACTAATTATGCTCTCTTGAAATTGGCTGGAGATGTTGAGAGCAACCCAGGTCCCATGGTGAGCAAGGGCGAGGAGGATAACATGGCCTCTCTCCCAGCGACACATGAGTTACACATCTTTGGCTCCATCAACGGTGTGGACTTTGACATGGTGGGTCAGGGCACCGGCAATCCAAATGATGGTTATGAGGAGTTAAACCTGAAGTCCACCAAGGGTGACCTCCAGTTCTCCCCCTGGATTCTGGTCCCTCATATCGGGTATGGCTTCCATCAGTACCTGCCCTACCCTGACGGGATGTCGCCTTTCCAGGCCGCCATGGTAGATGGCTCCGGATACCAAGTCCATCGCACAATGCAGTTTGAAGATGGTGCCTCCCTTACTGTTAACTACCGCTACACCTACGAGGGAAGCCACATCAAAGGAGAGGCCCAGGTGAAGGGGACTGGTTTCCCTGCTGACGGTCCTGTGATGACCAACTCGCTGACCGCTGCGGACTGGTGCAGGTCGAAGAAGACTTACCCCAACGACAAAACCATCATCAGTACCTTTAAGTGGAGTTACACCACTGGAAATGGCAAGCGCTACCGGAGCACTGCGCGGACCACCTACACCTTTGCCAAGCCAATGGCGGCTAACTATCTGAAGAACCAGCCGATGTACGTGTTCCGTAAGACGGAGCTCAAGCACTCCAAGACCGAGCTCAACTTCAAGGAGTGGCAAAAGGCCTTTACCGATGTGATGGGCATGGACGAGCTGTACAAGTAATGAATATTATCCCTAATACCTGCCACCCCACTCTTAATCAGTGGTGGAAGAACGGTCTCAGAACTGTTTGTTTCAATTGGCCATTTAAGTTTAGTAGTAAAAGACTGGTTAATGATAACAATGCATCGTAAAACCTTCAGAAGGAAAGGAGAATGTTTTGTGGACCACTTTGGTTTTCTTTTTTGCGTGTGGCAGTTTTAAGTTATTAGTTTTTAAAATCAGTACTTTTTAATGGAAACAACTTGACCAAAAATTTGTCACAGAATTTTGAGACCCATTAAAAAAGTTAAATGAGAAACCTGTGTGTTCCTTTGGTCAACACCGAGACATTTAGGTGAAAGACATCTAATTCTGGTTTTACGAATCTGGAAACTTCTTGAAAATGTAATTCTTGAGTTAACACTTCTGGGTGGAGAATAGGGTTGTTTTCCCCCCACATAATTGGAAGGGGAAGGAATATCATTTAAAGCTATGGGAGGGTTGCTTTGATTACAACACTGGAGAGAAATGCAGCATGTTGCTGATTGCC

**GAPDH-E2A-mNeonGreen (Left HA-E2A-mNeonGreen-Right HA)**

cccacctttctcatccaagactggctcctccctgccggggctgcgtgcaaccctggggttgggggttctggggactggctttcccataatttcctttcaaggtggggagggaggtagaggggtgatgtggggagtacgctgcagggcctcactccttttgcagACCACAGTCCATGCCATCACTGCCACCCAGAAGACTGTGGATGGCCCCTCCGGGAAACTGTGGCGTGATGGCCGCGGGGCTCTCCAGAACATCATCCCTGCCTCTACTGGCGCTGCCAAGGCTGTGGGCAAGGTCATCCCTGAGCTGAACGGGAAGCTCACTGGCATGGCCTTCCGTGTCCCCACTGCCAACGTGTCAGTGGTGGACCTGACCTGCCGTCTAGAAAAACCTGCCAAATATGATGACATCAAGAAGGTGGTGAAGCAGGCGTCGGAGGGCCCCCTCAAGGGCATCCTGGGCTACACTGAGCACCAGGTGGTCTCCTCTGACTTCAACAGCGACACCCACTCCTCCACCTTTGACGCTGGGGCTGGCATTGCCCTCAACGACCACTTTGTCAAGCTCATTTCCTGGTATGACAACGAATTTGGCTACAGCAACAGGGTGGTGGACCTCATGGCCCACATGGCCTCCAAGGAGCAGTGTACTAATTATGCTCTCTTGAAATTGGCTGGAGATGTTGAGAGCAACCCAGGTCCCATGGTGAGCAAGGGCGAGGAGGATAACATGGCCTCTCTCCCAGCGACACATGAGTTACACATCTTTGGCTCCATCAACGGTGTGGACTTTGACATGGTGGGTCAGGGCACCGGCAATCCAAATGATGGTTATGAGGAGTTAAACCTGAAGTCCACCAAGGGTGACCTCCAGTTCTCCCCCTGGATTCTGGTCCCTCATATCGGGTATGGCTTCCATCAGTACCTGCCCTACCCTGACGGGATGTCGCCTTTCCAGGCCGCCATGGTAGATGGCTCCGGATACCAAGTCCATCGCACAATGCAGTTTGAAGATGGTGCCTCCCTTACTGTTAACTACCGCTACACCTACGAGGGAAGCCACATCAAAGGAGAGGCCCAGGTGAAGGGGACTGGTTTCCCTGCTGACGGTCCTGTGATGACCAACTCGCTGACCGCTGCGGACTGGTGCAGGTCGAAGAAGACTTACCCCAACGACAAAACCATCATCAGTACCTTTAAGTGGAGTTACACCACTGGAAATGGCAAGCGCTACCGGAGCACTGCGCGGACCACCTACACCTTTGCCAAGCCAATGGCGGCTAACTATCTGAAGAACCAGCCGATGTACGTGTTCCGTAAGACGGAGCTCAAGCACTCCAAGACCGAGCTCAACTTCAAGGAGTGGCAAAAGGCCTTTACCGATGTGATGGGCATGGACGAGCTGTACAAGTAAGACCCCTGGACCACCAGCCCCAGCAAGAGCACAAGAGGAAGAGAGAGACCCTCACTGCTGGGGAGTCCCTGCCACACTCAGTCCCCCACCACACTGAATCTCCCCTCCTCACAGTTGCCATGTAGACCCCTTGAAGAGGGGAGGGGCCTAGGGAGCCGCACCTTGTCATGTACCATCAATAAAGTACCCTGTGCTCAACCAGTTACTTGTCCTGTCTTATTCTAGGGTCTGGGGCAGAGGGGAGGGAAGCTGGGCTTGTGTCAAGGTGAGACATTCTTGCTGGGGAGGGACCTGGTATGTTCTCCTCAGACTGAGGGTAGGGCCTCCAAACAGCCTTGCTTGCTTCGAGAACCATTTGCTTCCCGCTCAGACGTCTTGAGTGCTACAGGAAGCTGGCACCACTACTTCAGAGAACAAGGCCTTTTCCTCTCCTCGCTCCAGTCCTAGGCTATCTGCTGTTGGCCAAACATGGAAGAAGCTATTCTGTGGGC

**EEF1A1-E2A-mNeonGreen-Intron 7 (Left HA-Intron-E2A-mNeonGreen-Right HA)**

ggctgtaaacaaagttgaatttgagttgatagagtactgtctgccttcataggtatttagtatgctgtaaatatttttagGTATTGGTACTGTTCCTGTTGGCCGAGTGGAGACTGGTGTTCTCAAACCCGGTATGGTGGTCACCTTTGCTCCAGTCAACGTTACAACGGAAGTAAAATCTGTCGAAATGCACCATGAAGCTTTGAGTGAAGCTCTTCCTGGGGACAATGTGGGCTTCAATGTCAAGAATGTGTCTGTCAAGGATGTTCGTCGTGGCAACGTTGCTGGTGACAGCAAAAATGACCCACCAATGGAAGCAGCTGGCTTCACTGCTCAGgtaacaatttaaagtaacattaacttattgcagaggctaaagtcatttgagactttggatttgcactgaatgcaaatcttttttccaagGTGATTATCCTGAACCATCCAGGCCAAATAAGCGCCGGCTATGCCCCTGTATTGGATTGCCACACGGCTCACATTGCATGCAAGTTTGCTGAGCTGAAGGAAAAGATTGATCGCCGTTCTGGTAAAAAGCTGGAAGATGGCCCTAAATTCTTGAAGTCTGGTGATGCTGCCATTGTTGATATGGTTCCTGGCAAGCCCATGTGTGTTGAGAGCTTCTCAGACTATCCACCTTTGGgtaaggatgactacttaaatgtaaaaaagttgtgttaaagatgaaaaatacaactgaacaggtttaaacgcgtgggtaataattaactttttttttaatagGTCGCTTTGCTGTTCGTGATATGAGACAGACAGTTGCGGTGGGTGTCATCAAAGCAGTGGACAAGAAGGCTGCTGGAGCTGGCAAGGTCACCAAGTCTGCCCAGAAAGCTCAGAAGGCTAAACAGTGTACTAATTATGCTCTCTTGAAATTGGCTGGAGATGTTGAGAGCAACCCAGGTCCCATGGTGAGCAAGGGCGAGGAGGATAACATGGCCTCTCTCCCAGCGACACATGAGTTACACATCTTTGGCTCCATCAACGGTGTGGACTTTGACATGGTGGGTCAGGGCACCGGCAATCCAAATGATGGTTATGAGGAGTTAAACCTGAAGTCCACCAAGGGTGACCTCCAGTTCTCCCCCTGGATTCTGGTCCCTCATATCGGGTATGGCTTCCATCAGTACCTGCCCTACCCTGACGGGATGTCGCCTTTCCAGGCCGCCATGGTAGATGGCTCCGGATACCAAGTCCATCGCACAATGCAGTTTGAAGATGGTGCCTCCCTTACTGTTAACTACCGCTACACCTACGAGGGAAGCCACATCAAAGGAGAGGCCCAGGTGAAGGGGACTGGTTTCCCTGCTGACGGTCCTGTGATGACCAACTCGCTGACCGCTGCGGACTGGTGCAGGTCGAAGAAGACTTACCCCAACGACAAAACCATCATCAGTACCTTTAAGTGGAGTTACACCACTGGAAATGGCAAGCGCTACCGGAGCACTGCGCGGACCACCTACACCTTTGCCAAGCCAATGGCGGCTAACTATCTGAAGAACCAGCCGATGTACGTGTTCCGTAAGACGGAGCTCAAGCACTCCAAGACCGAGCTCAACTTCAAGGAGTGGCAAAAGGCCTTTACCGATGTGATGGGCATGGACGAGCTGTACAAGTAATGAATATTATCCCTAATACCTGCCACCCCACTCTTAATCAGTGGTGGAAGAACGGTCTCAGAACTGTTTGTTTCAATTGGCCATTTAAGTTTAGTAGTAAAAGACTGGTTAATGATAACAATGCATCGTAAAACCTTCAGAAGGAAAGGAGAATGTTTTGTGGACCACTTTGGTTTTCTTTTTTGCGTGTGGCAGTTTTAAGTTATTAGTTTTTAAAATCAGTACTTTTTAATGGAAACAACTTGACCAAAAATTTGTCACAGAATTTTGAGACCCATTAAAAAAGTTAAATGAGAAACCTGTGTGTTCCTTTGGTCAACACCGAGACATTTAGGTGAAAGACATCTAATTCTGGTTTTACGAATCTGGAAACTTCTTGAAAATGTAATTCTTGAGTTAACACTTCTGGGTGGAGAATAGGGTTGTTTTCCCCCCACATAATTGGAAGGGGAAGGAATATCATTTAAAGCTATGGGAGGGTTGCTTTGATTACAACACTGGAGAGAAATGCAGCATGTTGCTGATTGCC

**GAPDH-E2A-mNeonGreen-Intron 8 (Left HA-Intron-E2A-mNeonGreen-Right HA)**

ggctcccacctttctcatccaagactggctcctccctgccggggctgcgtgcaaccctggggttgggggttctggggactggctttcccataatttcctttcaaggtggggagggaggtagaggggtgatgtggggagtacgctgcagggcctcactccttttgcagACCACAGTCCATGCCATCACTGCCACCCAGAAGACTGTGGATGGCCCCTCCGGGAAACTGTGGCGTGATGGCCGCGGGGCTCTCCAGAACATCATCCCTGCCTCTACTGGCGCTGCCAAGGCTGTGGGCAAGGTCATCCCTGAGCTGAACGGGAAGCTCACTGGCATGGCCTTCCGTGTCCCCACTGCCAACGTGTCAGTGGTGGACCTGACCTGCCGTCTAGAAAAACCTGCCAAATATGATGACATCAAGAAGGTGGTGAAGCAGGCGTCGGAGGGCCCCCTCAAGGGCATCCTGGGCTACACTGAGCACCAGGTGGTCTCCTCTGACTTCAACAGCGACACCCACTCCTCCACCTTTGACGCTGGGGCTGGCATTGCCCTCAACGACCACTTTGTCAAGCTCATTTCCTGgtatgtggctggggccagagactggctcttgtttaaacgcgttgcagggtctggcgccctctggtggctggctcagaaaaagggccctgacaactcttttcatcttctagGTATGACAACGAATTTGGCTACAGCAACAGGGTGGTGGACCTCATGGCCCACATGGCCTCCAAGGAGCAGTGTACTAATTATGCTCTCTTGAAATTGGCTGGAGATGTTGAGAGCAACCCAGGTCCCATGGTGAGCAAGGGCGAGGAGGATAACATGGCCTCTCTCCCAGCGACACATGAGTTACACATCTTTGGCTCCATCAACGGTGTGGACTTTGACATGGTGGGTCAGGGCACCGGCAATCCAAATGATGGTTATGAGGAGTTAAACCTGAAGTCCACCAAGGGTGACCTCCAGTTCTCCCCCTGGATTCTGGTCCCTCATATCGGGTATGGCTTCCATCAGTACCTGCCCTACCCTGACGGGATGTCGCCTTTCCAGGCCGCCATGGTAGATGGCTCCGGATACCAAGTCCATCGCACAATGCAGTTTGAAGATGGTGCCTCCCTTACTGTTAACTACCGCTACACCTACGAGGGAAGCCACATCAAAGGAGAGGCCCAGGTGAAGGGGACTGGTTTCCCTGCTGACGGTCCTGTGATGACCAACTCGCTGACCGCTGCGGACTGGTGCAGGTCGAAGAAGACTTACCCCAACGACAAAACCATCATCAGTACCTTTAAGTGGAGTTACACCACTGGAAATGGCAAGCGCTACCGGAGCACTGCGCGGACCACCTACACCTTTGCCAAGCCAATGGCGGCTAACTATCTGAAGAACCAGCCGATGTACGTGTTCCGTAAGACGGAGCTCAAGCACTCCAAGACCGAGCTCAACTTCAAGGAGTGGCAAAAGGCCTTTACCGATGTGATGGGCATGGACGAGCTGTACAAGTAAGACCCCTGGACCACCAGCCCCAGCAAGAGCACAAGAGGAAGAGAGAGACCCTCACTGCTGGGGAGTCCCTGCCACACTCAGTCCCCCACCACACTGAATCTCCCCTCCTCACAGTTGCCATGTAGACCCCTTGAAGAGGGGAGGGGCCTAGGGAGCCGCACCTTGTCATGTACCATCAATAAAGTACCCTGTGCTCAACCAGTTACTTGTCCTGTCTTATTCTAGGGTCTGGGGCAGAGGGGAGGGAAGCTGGGCTTGTGTCAAGGTGAGACATTCTTGCTGGGGAGGGACCTGGTATGTTCTCCTCAGACTGAGGGTAGGGCCTCCAAACAGCCTTGCTTGCTTCGAGAACCATTTGCTTCCCGCTCAGACGTCTTGAGTGCTACAGGAAGCTGGCACCACTACTTCAGAGAACAAGGCCTTTTCCTCTCCTCGCTCCAGTCCTAGGCTATCTGCTGTTGGCCAAACATGGAAGAAGCTATTCTGTGGGCAGCCC

**OCT4-E2A-Crimson (Left HA-E2A-Crimson-Right HA)**

tcagggtcacaagggccctgcgtgctccctcactttgcttctcttttgactggcctcccccagGGAAGGTATTCAGCCAAACGACCATCTGCCGCTTTGAGGCTCTGCAGCTTAGCTTCAAGAACATGTGTAAGCTGCGGCCCTTGCTGCAGAAGTGGGTGGAGGAAGCTGACAACAATGAAAATCTTCAGGAGgtaagggtgggagggggatacccggggaccttccctttcttggcctaatttccattgcttccatcactggctcgtagctctccgtctttggtgcagtggttctcagtgggatggagtgaaattcctcagttctgctgggataaggtccagagccaacccttccaggatcctgccttttcacaccaccacctggctctgctgacacatctagtcacagacccctgtgatgctgttactcagcaagtccaaagcttgcccttgtcacccccttcccacctgcacagATATGCAAAGCAGAAACCCTCGTGCAGGCCCGAAAGAGAAAGCGAACCAGTATCGAGAACCGAGTGAGAGGCAACCTGGAGAATTTGTTCCTGCAGTGCCCGAAACCCACACTGCAGCAGATCAGCCACATCGCCCAGCAGCTTGGGCTCGAGAAGGATGTGGTCCGAGTGTGGTTCTGTAACCGGCGCCAGAAGGGCAAGCGATCAAGCAGCGACTATGCACAACGAGAGGATTTTGAGGCTGCTGGGTCTCCTTTCTCAGGGGGACCAGTGTCCTTTCCTCTGGCCCCAGGGCCCCATTTTGGTACCCCAGGCTATGGGAGCCCTCACTTCACTGCACTGTACTCCTCGGTCCCTTTCCCTGAGGGGGAAGCCTTTCCCCCTGTCTCCGTCACCACTCTGGGCTCTCCCATGCATTCAAACCAGTGTACTAATTATGCTCTCTTGAAATTGGCTGGAGATGTTGAGAGCAACCCAGGTCCCATGGATAGCACTGAGAACGTCATCAAGCCCTTCATGCGCTTCAAGGTGCACATGGAGGGCTCCGTGAACGGCCACGAGTTCGAGATCGAGGGCGTGGGCGAGGGCAAGCCCTACGAGGGCACCCAGACCGCCAAGCTGCAAGTGACCAAGGGCGGCCCCCTGCCCTTCGCCTGGGACATCCTGTCCCCCCAGTTCTTCTACGGCTCCAAGGCGTACATCAAGCACCCCGCCGACATCCCCGACTACCTCAAGCAGTCCTTCCCCGAGGGCTTCAAGTGGGAGCGCGTGATGAACTTCGAGGACGGCGGCGTGGTGACCGTGACCCAGGACTCCTCCCTGCAGGACGGCACCCTCATCTACCACGTGAAGTTCATCGGCGTGAACTTCCCCTCCGACGGCCCCGTAATGCAGAAGAAGACTCTGGGCTGGGAGCCCTCCACTGAGCGCAACTACCCCCGCGACGGCGTGCTGAAGGGCGAGAACCACATGGCGCTGAAGCTGAAGGGCGGCGGCCACTACCTGTGTGAGTTCAAGTCCATCTACATGGCCAAGAAGCCCGTGAAGCTGCCCGGCTACCACTACGTGGACTACAAGCTCGACATCACCTCCCACAACGAGGACTACACCGTGGTGGAGCAGTACGAGCGCGCCGAGGCCCGCCACCACCTGTTCCAGTAGGGTGCCTGCCCTTCTAGGAATGGGGGACAGGGGGAGGGGAGGAGCTAGGGAAAGAAAACCTGGAGTTTGTGCCAGGGTTTTTGGGATTAAGTTCTTCATTCACTAAGGAAGGAATTGGGAACACAAAGGGTGGGGGCAGGGGAGTTTGGGGCAACTGGTTGGAGGGAAGGTGAAGTTCAATGATGCTCTTGATTTTAATCCCACATCATGTATCACTTTTTTCTTAAATAAAGAAGCCTGGGACACAGTAGATAGACACACTTATCTTGGTTTGTCCTTCAGTTACTGAGGTAGGGATGGGAATATCCAATGCTCATACCCAAGTGACCCTGAAACTAAGGTGCCATTTACACTCCTTAAGGTCACACAACATCAGAGGGAGAGCTGGGATTGCAGCCAAGTTTATTTGTACAGGGCCCTGTGATAGGCTAGTTCCCAAAAGCCTGTGATGCAAGAACTTTTGCCCATAGACTCAGTCACCATGTAGCTGTTACCTGTTCAGAGCTGGCTTTTTGCTTTCCCACCCTACTCTGGAATTCTTAAATGGCTTTATACTTAGAAATCATCTTATTTCTGTTGAACCTAGATCA

**OCT4-E2A-Crimson-Intron 4 (Left HA-Intron-E2A-Crimson-Right HA)**

tcagggtcacaagggccctgcgtgctccctcactttgcttctcttttgactggcctcccccagGGAAGGTATTCAGCCAAACGACCATCTGCCGCTTTGAGGCTCTGCAGCTTAGCTTCAAGAACATGTGTAAGCTGCGGCCCTTGCTGCAGAAGTGGGTGGAGGAAGCTGACAACAATGAAAATCTTCAGGAGgtaagggtgggagggggatacccggggaccttccctttcttggcctaatttccattgcttccatcactggctcgtagctctccgtctttggtgcagtggttctcagtgggatggagtgaaattcctcagttctgctgggataaggtccagagccaacccttccaggatcctgccttttcacaccaccacctggctctgctgacacatctagtcacagacccctgtgatgctgttactcagcaagtccaaagcttgCCCTTGTCACCCCCTTCCCACCTGCACAGATATGCAAAGCAGAAACCCTCGTGCAGGCCCGAAAGAGAAAGCGAACCAGTATCGAGAACCGAGTGAGAGGCAACCTGGAGAATTTGTTCCTGCAGTGCCCGAAACCCACACTGCAGCAGATCAGCCACATCGCCCAGCAGCTTGGGCTCGAGAAGGATgtgagtgccatgtcgtttaaacgcgtcgggctccatctctttcccctgtcaccacctcgctttccctagctctggctcctccaactgctctagggctgttggctttggacagaatgtccaagcagtcaggcctgtctcagctcattctctaatgtcctcctctaactgctctagggctgttggctttggatagaatgtccaagcagagtcaggcccgtctcagctcattgtctaatgtcattctcctttctgtcattcacttgcagGTGGTCCGAGTGTGGTTCTGTAACCGGCGCCAGAAGGGCAAGCGATCAAGCAGCGACTATGCACAACGAGAGGATTTTGAGGCTGCTGGGTCTCCTTTCTCAGGGGGACCAGTGTCCTTTCCTCTGGCCCCAGGGCCCCATTTTGGTACCCCAGGCTATGGGAGCCCTCACTTCACTGCACTGTACTCCTCGGTCCCTTTCCCTGAGGGGGAAGCCTTTCCCCCTGTCTCCGTCACCACTCTGGGCTCTCCCATGCATTCAAACCAGTGTACTAATTATGCTCTCTTGAAATTGGCTGGAGATGTTGAGAGCAACCCAGGTCCCATGGATAGCACTGAGAACGTCATCAAGCCCTTCATGCGCTTCAAGGTGCACATGGAGGGCTCCGTGAACGGCCACGAGTTCGAGATCGAGGGCGTGGGCGAGGGCAAGCCCTACGAGGGCACCCAGACCGCCAAGCTGCAAGTGACCAAGGGCGGCCCCCTGCCCTTCGCCTGGGACATCCTGTCCCCCCAGTTCTTCTACGGCTCCAAGGCGTACATCAAGCACCCCGCCGACATCCCCGACTACCTCAAGCAGTCCTTCCCCGAGGGCTTCAAGTGGGAGCGCGTGATGAACTTCGAGGACGGCGGCGTGGTGACCGTGACCCAGGACTCCTCCCTGCAGGACGGCACCCTCATCTACCACGTGAAGTTCATCGGCGTGAACTTCCCCTCCGACGGCCCCGTAATGCAGAAGAAGACTCTGGGCTGGGAGCCCTCCACTGAGCGCAACTACCCCCGCGACGGCGTGCTGAAGGGCGAGAACCACATGGCGCTGAAGCTGAAGGGCGGCGGCCACTACCTGTGTGAGTTCAAGTCCATCTACATGGCCAAGAAGCCCGTGAAGCTGCCCGGCTACCACTACGTGGACTACAAGCTCGACATCACCTCCCACAACGAGGACTACACCGTGGTGGAGCAGTACGAGCGCGCCGAGGCCCGCCACCACCTGTTCCAGTAGGGTGCCTGCCCTTCTAGGAATGGGGGACAGGGGGAGGGGAGGAGCTAGGGAAAGAAAACCTGGAGTTTGTGCCAGGGTTTTTGGGATTAAGTTCTTCATTCACTAAGGAAGGAATTGGGAACACAAAGGGTGGGGGCAGGGGAGTTTGGGGCAACTGGTTGGAGGGAAGGTGAAGTTCAATGATGCTCTTGATTTTAATCCCACATCATGTATCACTTTTTTCTTAAATAAAGAAGCCTGGGACACAGTAGATAGACACACTTATCTTGGTTTGTCCTTCAGTTACTGAGGTAGGGATGGGAATATCCAATGCTCATACCCAAGTGACCCTGAAACTAAGGTGCCATTTACACTCCTTAAGGTCACACAACATCAGAGGGAGAGCTGGGATTGCAGCCAAGTTTATTTGTACAGGGCCCTGTGATAGGCTAGTTCCCAAAAGCCTGTGATGCAAGAACTTTTGCCCATAGACTCAGTCACCATGTAGCTGTTACCTGTTCAGAGCTGGCTTTTTGCTTTCCCACCCTACTCTGGAATTCTTAAATGGCTTTATACTTAGAAATCATCTTATTTCTGTTGAACCTAGATCA

**OCT4-E2A-Crimson-EEF1A1 intron 7 (Left HA-Intron-E2A-Crimson-Right HA)**

tcagggtcacaagggccctgcgtgctccctcactttgcttctcttttgactggcctcccccagGGAAGGTATTCAGCCAAACGACCATCTGCCGCTTTGAGGCTCTGCAGCTTAGCTTCAAGAACATGTGTAAGCTGCGGCCCTTGCTGCAGAAGTGGGTGGAGGAAGCTGACAACAATGAAAATCTTCAGGAGgtaagggtgggagggggatacccggggaccttccctttcttggcctaatttccattgcttccatcactggctcgtagctctccgtctttggtgcagtggttctcagtgggatggagtgaaattcctcagttctgctgggataaggtccagagccaacccttccaggatcctgccttttcacaccaccacctggctctgctgacacatctagtcacagacccctgtgatgctgttactcagcaagtccaaagcttgCCCTTGTCACCCCCTTCCCACCTGCACAGATATGCAAAGCAGAAACCCTCGTGCAGGCCCGAAAGAGAAAGCGAACCAGTATCGAGAACCGAGTGAGAGGCAACCTGGAGAATTTGTTCCTGCAGTGCCCGAAACCCACACTGCAGCAGATCAGCCACATCGCCCAGCAGCTTGGGCTCGAGAAGGATgtaaggatgactacttaaatgtaaaaaagttgtgttaaagatgaaaaatacaactgaacaggtttaaacgcgtgggtaataattaactttttttttaatagGTGGTCCGAGTGTGGTTCTGTAACCGGCGCCAGAAGGGCAAGCGATCAAGCAGCGACTATGCACAACGAGAGGATTTTGAGGCTGCTGGGTCTCCTTTCTCAGGGGGACCAGTGTCCTTTCCTCTGGCCCCAGGGCCCCATTTTGGTACCCCAGGCTATGGGAGCCCTCACTTCACTGCACTGTACTCCTCGGTCCCTTTCCCTGAGGGGGAAGCCTTTCCCCCTGTCTCCGTCACCACTCTGGGCTCTCCCATGCATTCAAACCAGTGTACTAATTATGCTCTCTTGAAATTGGCTGGAGATGTTGAGAGCAACCCAGGTCCCATGGATAGCACTGAGAACGTCATCAAGCCCTTCATGCGCTTCAAGGTGCACATGGAGGGCTCCGTGAACGGCCACGAGTTCGAGATCGAGGGCGTGGGCGAGGGCAAGCCCTACGAGGGCACCCAGACCGCCAAGCTGCAAGTGACCAAGGGCGGCCCCCTGCCCTTCGCCTGGGACATCCTGTCCCCCCAGTTCTTCTACGGCTCCAAGGCGTACATCAAGCACCCCGCCGACATCCCCGACTACCTCAAGCAGTCCTTCCCCGAGGGCTTCAAGTGGGAGCGCGTGATGAACTTCGAGGACGGCGGCGTGGTGACCGTGACCCAGGACTCCTCCCTGCAGGACGGCACCCTCATCTACCACGTGAAGTTCATCGGCGTGAACTTCCCCTCCGACGGCCCCGTAATGCAGAAGAAGACTCTGGGCTGGGAGCCCTCCACTGAGCGCAACTACCCCCGCGACGGCGTGCTGAAGGGCGAGAACCACATGGCGCTGAAGCTGAAGGGCGGCGGCCACTACCTGTGTGAGTTCAAGTCCATCTACATGGCCAAGAAGCCCGTGAAGCTGCCCGGCTACCACTACGTGGACTACAAGCTCGACATCACCTCCCACAACGAGGACTACACCGTGGTGGAGCAGTACGAGCGCGCCGAGGCCCGCCACCACCTGTTCCAGTAGGGTGCCTGCCCTTCTAGGAATGGGGGACAGGGGGAGGGGAGGAGCTAGGGAAAGAAAACCTGGAGTTTGTGCCAGGGTTTTTGGGATTAAGTTCTTCATTCACTAAGGAAGGAATTGGGAACACAAAGGGTGGGGGCAGGGGAGTTTGGGGCAACTGGTTGGAGGGAAGGTGAAGTTCAATGATGCTCTTGATTTTAATCCCACATCATGTATCACTTTTTTCTTAAATAAAGAAGCCTGGGACACAGTAGATAGACACACTTATCTTGGTTTGTCCTTCAGTTACTGAGGTAGGGATGGGAATATCCAATGCTCATACCCAAGTGACCCTGAAACTAAGGTGCCATTTACACTCCTTAAGGTCACACAACATCAGAGGGAGAGCTGGGATTGCAGCCAAGTTTATTTGTACAGGGCCCTGTGATAGGCTAGTTCCCAAAAGCCTGTGATGCAAGAACTTTTGCCCATAGACTCAGTCACCATGTAGCTGTTACCTGTTCAGAGCTGGCTTTTTGCTTTCCCACCCTACTCTGGAATTCTTAAATGGCTTTATACTTAGAAATCATCTTATTTCTGTTGAACCTAGATCA
